# Serum IgG2 levels are specifically associated with whole-body insulin-mediated glucose disposal in non-diabetic offspring of type 2 diabetic individuals: a cross-sectional study

**DOI:** 10.1038/s41598-018-32108-8

**Published:** 2018-09-11

**Authors:** Teresa Vanessa Fiorentino, Elena Succurro, Franco Arturi, Aida Giancotti, Cinzia Peronace, Angela Quirino, Franz Sesti, Francesco Andreozzi, Marta Letizia Hribal, Francesco Perticone, Alfredo Focà, Giorgio Sesti

**Affiliations:** 1 Department of Medical and Surgical Sciences, University Magna Graecia of Catanzaro, Viale Europa, Catanzaro, 88100 Italy; 2 Department of Health Sciences, University Magna Graecia of Catanzaro, Viale Europa, 88100 Catanzaro, Italy; 3 Department of Experimental Medicine, Sapienza University of Rome, Rome, 00161 Italy

## Abstract

Preclinical studies suggested that IgG2c isotype may specifically impair skeletal muscle insulin sensitivity in mice. In this study we investigated the association between serum levels of the four IgG subclasses and insulin sensitivity in non-diabetic individuals. Total IgG, IgG1, IgG2, IgG3 and IgG4 levels were measured in 262 subjects. Whole-body insulin sensitivity was assessed by euglycemic hyperinsulinemic clamp. IgG2 levels were positively correlated with BMI, waist circumference, 2-h post-load glucose levels and complement C3. Serum IgG2, but not IgG1, IgG3 and IgG4 levels were negatively correlated with whole-body insulin sensitivity (r = −0.17; P = 0.003) and muscle insulin sensitivity index (r = −0.16; P = 0.03) after adjustment for age and gender. No significant correlation was found between IgG2 levels and hepatic insulin resistance assessed by HOMA-IR and liver IR index. In a multivariable regression analysis including variables known to affect insulin sensitivity such as age, gender, BMI, smoking, lipids, inflammatory markers, fasting and 2-h post-load glucose levels, IgG2 levels were independently associated with insulin-stimulated glucose disposal (β = −0.115, 95% CI: −0.541 to −0.024; P = 0.03). These data demonstrate the independent association between higher levels of IgG2 and decreased whole-body insulin sensitivity, thus confirming in humans the animal-based evidence indicating the pathogenic role of IgG2 in insulin resistance.

## Introduction

Observational studies have solidified the concept that low-grade inflammation plays a pathophysiological role in insulin resistance associated with type 2 diabetes mellitus^1–7^. Markers of inflammation have been extensively studied in these metabolic conditions, with convincing evidence showing that elevated concentrations of C reactive protein, fibrinogen, complement C3, and white blood cells count are predictors of type 2 diabetes. In addition to these reliable markers of inflammation, widely used in clinical practice, also immunoglobulin G (IgG) level, an indicator of adaptive immune system activation, has been associated with obesity and type 2 diabetes in cross-sectional and prospective studies^8–10^. Experimental studies in mice have suggested a role of B cells in the pathogenesis of insulin resistance associated with diet-induced obesity^11,12^. In particular, it has been shown that transfer of proinflammatory IgG2c isotype purified from sera of diet-induced obese mice caused insulin resistance and glucose intolerance in the recipient rodents^11^. More recently, it has been reported that IgG2c isotype, but not IgG1 or IgG2b, selectively caused skeletal muscle insulin resistance in diet-induced obese mice by activating the IgG receptor FcγRIIB in endothelium^12^. The activation of FcγRIIB by IgG2c isotype from diet-induced obese mice, in turn, impaired endothelial cell insulin transcytosis, resulting in reduced insulin delivery to skeletal muscle and impairment in muscle glucose disposal^12^. Furthermore, IgG isolated from individuals with type 2 diabetes caused glucose intolerance and insulin resistance in IgG-deficient mice via FcγRIIB, indicating that similar processes may be effective in humans^12^. Prompted by the need to better understand the pathophysiological role of human IgG isotypes in glucose homeostasis disorders, we sought to determine their potential role in insulin resistance. To address this issue, we assessed human serum IgG isotypes (IgG1, IgG2, IgG3, and IgG4) in non-diabetic offspring of type 2 diabetic individuals and correlated their concentration with insulin sensitivity assessed using the euglycemic hyperinsulinemic clamp.

## Results

Clinical features and biochemical findings of the study group stratified by gender are shown in Table 1. Men were more likely to be current smokers and exhibited a worse cardio-metabolic risk profile including higher waist circumference values, blood pressure, triglycerides and fasting plasma glucose levels, and lower HDL cholesterol, muscle insulin sensitivity index and insulin-stimulated glucose disposal, assessed by euglycemic-hyperinsulinemic clamp. No gender-specific differences were observed in the other indexes of insulin sensitivity either based on fasting measurements such as QUICKI, and homeostasis model assessment insulin resistance (HOMA-IR) or derived by OGTT including liver insulin resistance (liver IR) index, glucose_0–30_ (AUC) × insulin_0–30_ (AUC) index, Stumvoll Insulin Sensitivity Index (ISI), Gutt’s ISI_0,120_, and Matsuda index. Additionally, no gender-specific differences were observed in total IgG or IgG isotypes levels except for IgG4, which were higher in men (Table 1).

**Table 1:** Clinical characteristics of the study group.

Variables	Overall cohort	Men	Women	*P* value
Number	262	137	125	—
Age (*yrs*)	39 ± 9	40 ± 10	38 ± 9	0.43
BMI (*kg*/*m*^2^)	30.0 ± 6.2	30.0 ± 5.0	30.1 ± 7.3	0.85
Normal weight/overweight/obese (*n*)	56/83/123	20/51/66	36/32/57	0.01
Waist circumference (*cm*)	99 ± 14	101 ± 12	96 ± 14	0.002
Fat body mass (%)	31 ± 9	27 ± 7	36 ± 8	<0.0001
Fat free mass (%)	69 ± 9	73 ± 7	64 ± 8	<0.0001
Systolic blood pressure (*mmHg*)	127 ± 15	131 ± 12	124 ± 17	<0.0001
Diastolic blood pressure (*mmHg*)	81 ± 11	84 ± 10	78 ± 11	<0.0001
Total Cholesterol (*mg*/*dl*)	195 ± 39	197 ± 37	192 ± 40	0.23
HDL Cholesterol (*mg*/*dl*)	50 ± 14	44 ± 11	56 ± 13	<0.0001
Triglycerides (*mg*/*dl*)	119 ± 69	137 ± 75	99 ± 55	<0.0001
Fasting Glucose (*mg*/*dl*)	90 ± 9	92 ± 9	88 ± 9	0.01
2-h glucose (*mg*/*dl*)	114 ± 26	113 ± 24	115 ± 27	0.44
Fasting insulin (*µU*/*ml*)	13.7 ± 8.8	14.4 ± 9.4	12.9 ± 8.0	0.19
HbA1c (%) [*mmol*/*mol*]	5.4 ± 0.3 [36 mmol/mol]	5.4 ± 0.3 [36 mmol/mol]	5.4 ± 0.3 [36 mmol/mol]	0.31
Total IgG (*mg*/*ml*)	11.4 ± 2.6	11.2 ± 2.6	11.7 ± 2.6	0.09
IgG1 (*mg*/*ml*)	6.5 ± 1.7	6.4 ± 1.9	6.6 ± 1.6	0.40
IgG2 (*mg*/*ml*)	4.0 ± 1.5	3.9 ± 1.3	4.1 ± 1.7	0.15
IgG3 (*mg*/*ml*)	0.40 ± 0.20	0.38 ± 0.17	0.42 ± 0.22	0.09
IgG4 (*mg*/*ml*)	0.65 ± 0.58	0.75 ± 0.17	0.42 ± 0.22	0.003
hsCRP (*mg*/*l*)	3.1 ± 4.2	2.9 ± 4.7	3.4 ± 3.6	0.22
Complement C3 (*g*/*l*)	1.19 ± 0.26	1.19 ± 0.23	1.18 ± 0.29	0.70
White blood cell count (*cell*/*mm*^3^)	7068 ± 1966	7205 ± 2033	6919 ± 1887	0.24
Insulin-stimulated glucose disposal (*mg* * *min*^−1^ * *kg FFM* ^−1^)	7.1 ± 3.7	6.4 ± 3.2	7.8 ± 4.1	0.004
HOMA-IR	3.0 ± 2.0	3.2 ± 2.1	2.8 ± 1.8	0.07
Liver IR index	2.97 ± 0.40	2.99 ± 0.41	2.96 ± 0.39	0.59
QUICKI	0.25 ± 0.04	0.25 ± 0.04	0.26 ± 0.04	0.10
Glucose_0–30_ (AUC) × insulin_0–30_ (AUC)	35.0 ± 23.7	36.7 ± 22.3	33.1 ± 25.2	0.07
Stumvoll ISI_OGTT_	0.061 ± 0.058	0.059 ± 0.068	0.063 ± 0.048	0.21
Matsuda index	74 ± 48	71 ± 49	78 ± 46	0.14
Gutt’s ISI_0,120_	19.2 ± 7.1	19.3 ± 7.4	19.1 ± 6.7	0.86
Muscle insulin sensitivity index	1.19 ± 1.12	1.05 ± 0.99	1.29 ± 1.21	0.03
NGT/IFG/IGT/combo IFG + IGT (*n*)	200/21/30/11	100/17/12/8	100/4/18/3	0.01
Smoking status (never smokers/current smokers/ex-smokers)	164/66/32	74/41/22	90/35/10	0.009

Data are means ± SD. Triglycerides, fasting insulin, HOMA-IR index, glucose_0–30_ (AUC) × insulin_0–30_ (AUC), Matsuda, Gutt’s ISI_0,120_, Stumvoll ISI_OGTT_, Muscle insulin sensitivity index, and hsCRP levels were log transformed for statistical analysis, but values in the table represent a back transformation to the original scale. Differences between means were compared using unpaired Student’s *t* test. Categorical variables were compared by χ^2^ test. NGT = normal glucose tolerance; IGT = impaired glucose tolerance; IFG = impaired fasting glucose; BMI = body mass index; hsCRP = high sensitivity C reactive protein; HbA1c = glycated hemoglobin; HDL = high density lipoprotein; HOMA-IR = homeostasis model assessment insulin resistance; Liver IR index = liver insulin resistance index.

Univariate correlations between circulating IgG isotypes levels and anthropometric and metabolic variables are shown in Table 2. After adjusting for age and gender, only IgG2 levels exhibited a significant correlation with anthropometric and metabolic traits with the exception of IgG1, which displayed an inverse relationship with percentage of fat free mass. Particularly, IgG2 levels were positively correlated with BMI, waist circumference, 2-h post-load glucose levels and complement C3. Serum IgG2, but not IgG1, IgG3, and IgG4 levels were negatively correlated with insulin-stimulated glucose disposal, and with the OGTT-derived insulin sensitivity indexes Stumvoll ISI, Matsuda, Gutt’s ISI_0,120_, and muscle insulin sensitivity index (Table 2). No significant association between IgG2 concentrations and hepatic insulin sensitivity estimated by HOMA-IR, liver IR and glucose_0–30_ (AUC) × insulin_0–30_ (AUC) index was observed. The correlation between IgG2 levels and insulin-stimulated glucose disposal remained significant after further adjusting for BMI in addition to gender and age (*P* = 0.005). By contrast, the correlations between IgG2 levels and 2-h post-load glucose or complement C3 levels were no longer statistically significant after adjustment for BMI. Furthermore, the negative relationship between IgG2 levels and insulin-stimulated glucose disposal was statistically significant in the subgroup of subjects with normal glucose tolerance (n = 200) as well as in the small subgroup of individuals with impaired glucose tolerance and/or impaired fasting glucose (n = 62) (Suppl. Tables 1 and 2).

**Table 2:** Age and gender adjusted univariate correlations between IgG isotypes levels and anthropometric and metabolic variables.

	Total IgG (*mg*/*ml*)		IgG1 (*mg*/*ml*)		IgG2 (*mg*/*ml*)		IgG3 (*mg*/*ml*)		IgG4 (*mg*/*ml*)	
	Pearson’s correlation coefficient (*r*)	*P* value	Pearson’s correlation coefficient (*r*)	*P* value	Pearson’s correlation coefficient (*r*)	*P* value	Pearson’s correlation coefficient (*r*)	*P* value	Pearson’s correlation coefficient (*r*)	*P* value
Age (*yrs*)	−0.01	0.38*	−0.07	0.26*	0.03	0.29*	0.01	0.95*	−0.05	0.37*
BMI (*kg*/*m*^2^)	0.09	0.07	0.11	0.07	**0.11**	**0.04**	−0.04	0.48	0.03	0.58
Waist circumference (*cm*)	0.06	0.18	0.09	0.14	**0.10**	**0.05**	−0.05	0.36	0.01	0.81
Fat body mass (%)	0.08	0.09	0.12	0.057	0.04	0.28	−0.06	0.28	0.05	0.40
Fat free mass (%)	−0.09	0.06	**−0.12**	**0.04**	−0.02	0.36	0.06	0.30	−0.03	0.63
Systolic blood pressure (*mmHg*)	0.04	0.25	0.01	0.82	0.03	0.31	0.01	0.81	0.03	0.58
Diastolic blood pressure (*mmHg*)	0.10	0.04	0.07	0.23	0.02	0.35	0.04	0.51	0.12	0.05
Total Cholesterol (*mg*/*dl*)	−0.01	0.40	0.03	0.66	−0.01	0.43	0.04	0.52	0.03	0.64
HDL Cholesterol (*mg*/*dl*)	−0.09	0.07	0.08	0.16	−0.09	0.08	−0.10	0.09	−0.06	0.32
Triglycerides (*mg*/*dl*)	0.05	0.21	0.08	0.18	0.07	0.12	0.12	0.06	0.06	0.28
Fasting Glucose (*mg*/*dl*)	−0.01	0.48	−0.04	0.55	0.05	0.20	−0.08	0.18	−0.02	0.75
2-h glucose (*mg*/*dl*)	0.01	0.40	−0.03	0.62	**0.14**	**0.01**	−0.02	0.71	−0.05	0.36
HbA1c (%) [*mmol*/*mol*]	−0.09	0.15	−0.04	0.68	0.03	0.38	0.04	0.61	0.05	0.57
hsCRP (*mg*/*l*)	0.05	0.20	0.02	0.75	0.10	0.06	−0.05	0.40	0.01	0.79
Complement C3 (*g*/*l*)	0.19	0.004	0.10	0.14	**0.12**	**0.04**	0.06	0.39	0.01	0.89
White blood cell count (*cell*/*mm*^3^)	0.01	0.47	0.01	0.84	0.06	0.16	0.01	0.83	0.02	0.73
Insulin-stimulated glucose disposal (*mg* * *min*^−1^ * *kg FFM*^−1^)	−0.07	0.28	−0.02	0.76	**−0.17**	**0.003**	−0.04	0.44	−0.02	0.76
HOMA-IR	0.11	0.07	0.07	0.24	0.11	0.07	0.01	0.83	0.01	0.98
Liver IR index	0.10	0.11	0.11	0.09	0.09	0.14	0.09	0.08	−0.02	0.78
QUICKI index	−0.06	0.17	−0.02	0.37	−0.09	0.07	0.03	0.29	0.01	0.44
Glucose_0–30_ (AUC) × insulin_0–30_ (AUC) index	0.07	0.31	0.09	0.19	0.04	0.60	0.09	0.16	−0.04	0.47
Stumvoll ISI_OGTT_	−0.09	0.18	−0.07	0.32	**−0.19**	**0.007**	0.003	0.96	−0.03	0.69
Matsuda index	−0.10	0.07	−0.06	0.18	**−0.14**	**0.02**	−0.04	0.27	−0.01	0.48
Gutt’s ISI_0,120_	−0.09	0.09	−0.03	0.34	**−0.17**	**0.006**	−0.02	0.39	0.02	0.37
Muscle insulin sensitivity index	**−0.16**	**0.03**	−0.06	0.39	**−0.16**	**0.03**	−0.13	0.09	0.04	0.58

*P values refer to results after analyses with adjustment for gender. Triglycerides, HOMA-IR index, glucose_0–30_ (AUC) × insulin_0–30_ (AUC), Stumvoll ISI_OGTT_, Matsuda, Gutt’s ISI_0,120_, Muscle insulin sensitivity index, and hsCRP levels were log transformed for statistical analysis.

BMI = body mass index; HbA1c = glycated hemoglobin; hsCRP = high sensitivity C reactive protein; HDL = high density lipoprotein; HOMA-IR = homeostasis model assessment insulin resistance; Liver IR index = liver insulin resistance index.

In order to confirm the specific association between insulin-stimulated glucose disposal and IgG2 levels we subdivided the whole study population in tertiles of insulin-stimulated glucose disposal. After adjusting for age and gender serum IgG2 levels, but not IgG1, IgG3, and IgG4 levels progressively decreased in the intermediate and highest tertiles of insulin-stimulated glucose disposal in comparison to the lowest tertile (Table 3).

**Table 3:** IgG isotypes levels of the study subjects stratified according to tertiles of insulin-stimulated glucose disposal.

	Tertile 1	Tertile 2	Tertile 3	*P value adjusted for age and gender*
Male/female	49/38	52/36	36/51	0.04
Age (*yrs*)	38 ± 10	40 ± 10	39 ± 9	0.47
BMI (*kg*/*m*^2^)	32.1 ± 6.4	30.4 ± 5.2	27.5 ± 6.1	<0.0001
Total IgG (*mg*/*ml*)	11.4 ± 2.5	11.4 ± 2.7	11.2 ± 2.5	0.87
IgG1 (*mg*/*ml*)	6.4 ± 1.6	6.5 ± 1.6	6.5 ± 1.8	0.79
IgG2 (*mg*/*ml*)	4.2 ± 1.5	4.0 ± 1.6	3.7 ± 1.3	0.04
IgG3 (*mg*/*ml*)	0.39 ± 0.21	0.41 ± 0.20	0.39 ± 0.18	0.75
IgG4 (*mg*/*ml*)	0.53 ± 0.06	0.69 ± 0.07	0.49 ± 0.05	0.42
Insulin-stimulated glucose disposal (*mg* * *min*^−1^ * *kg FFM* ^−1^)	3.4 ± 0.9	6.5 ± 0.8	11.2 ± 3.0	<0.0001

Data are means ± SD. Comparisons between the three groups were performed using a general linear model for multiple comparisons. BMI = body mass index.

Next, we built three models of multivariable regression analysis including variables known to affect insulin sensitivity to evaluate the independent contribution of IgG2 levels to insulin-stimulated glucose disposal (Table 4). Comparison of standardized coefficients allowed the determination of the relative strength of each trait association with insulin-stimulated glucose disposal. We found that IgG2 levels were associated with insulin-stimulated glucose disposal independently of age, gender, and BMI (Table 4, model 1). The association between IgG2 levels and insulin-stimulated glucose disposal remained significant when smoking status, blood pressure, total and HDL cholesterol, triglycerides, fasting and 2-h post-load glucose concentrations were included in the model in addition to age, gender, and BMI (Table 4, model 2). Similar results were obtained when waist circumference replaced BMI in the regression model (Table 4, model 3). In a full adjusted model including hsCRP, white blood cells count, and complement C3 in addition to variables analyzed in the model 2, IgG2 levels were independently associated with insulin-stimulated glucose disposal (β = −0.115, *P* = 0.03), with the model explaining 32.9% of variation (R^2^ = 0.324) of insulin-stimulated glucose disposal.

**Table 4:** Multiple regression analyses evaluating insulin-stimulated glucose disposal as dependent variable in four models of increasing complexity.

	Independent contributors	Standardized Coefficient β (95% CI)	*P*
**Model 1** includes IgG2, gender, age, and BMI.	BMI	−0.353 (−0.278 to −0.145)	<0.0001
	Gender (male)	−0.199 (−2.319 to −0.649)	0.001
	IgG2	−0.141 (−0.622 to −0.071)	0.01
**Model 2** includes IgG2, gender, age, BMI, smoking status, blood pressure, total and HDL cholesterol, triglycerides, fasting and 2 h post-load plasma glucose.	2 h post-load glucose	−0.240 (−0.052 to −0.020)	<0.0001
	BMI	−0.220 (−0.193 to −0.055)	<0.0001
	Age	0.136 (0.001 to 0.094)	0.03
	IgG2	−0.120 (−0.535 to −0.012)	0.02
**Model 3** includes IgG2, gender, age, waist circumference, smoking status, blood pressure, total and HDL cholesterol, triglycerides, fasting and 2 h post-load plasma glucose.	2 h post-load glucose	−0.240 (−0.050 to −0.018)	<0.0001
	Waist circumference	−0.201 (−0.084 to −0.023)	0.001
	Age	0.146 (0.007 to 0.101)	0.03
	IgG2	−0.130 (−0.563 to −0.038)	0.03
**Model 4** IgG2, gender, age, BMI, smoking status, blood pressure, total and HDL cholesterol, triglycerides, fasting and 2 h post-load plasma glucose, hsCRP, white blood cell count, and complement C3.	2 h post-load glucose	−0.217 (−0.047 to −0.015)	<0.0001
	BMI	−0.153 (−0.169 to −0.014)	0.02
	hsCRP	−0.138 (−0.971 to −0.011)	0.04
	IgG2	−0.115 (−0.541 to −0.024)	0.03

Linear regression analysis in models including insulin-stimulated insulin sensitivity as dependent variables and gender, age, BMI, waist circumference, smoking status, systolic and diastolic blood pressure, total and HDL cholesterol, triglycerides, fasting and 2 h post-load plasma glucose, hsCRP, white blood cell count, and complement C3 as independent contributors.

BMI = body mass index; hsCRP = high sensitivity C reactive protein; HDL = high density lipoprotein.

## Discussion

Prior cross-sectional and prospective studies have shown that total serum IgG levels, a nonspecific marker of the adaptive immune system activation, are associated with obesity and type 2 diabetes^8–10^. Additionally, animal-based studies have suggested that IgG2c isotype, but not IgG1 or IgG2b, may have a role in causing a selective state of muscle insulin resistance in diet-induced obese mice^11,12^. These observations provided the rationale to investigate the relationship between serum IgG1, IgG2, IgG3, and IgG4 isotypes and insulin sensitivity in humans. In this cross-sectional study including nondiabetic offspring of type 2 diabetic individuals with a wide range of BMI, we found that in univariate analysis serum concentrations of IgG2, but not IgG1, IgG3, or IgG4, were inversely associated with whole-body insulin-stimulated glucose disposal. This association remained statistically significant in multivariate analysis after adjustments for potential confounders such as indexes of adiposity, lipids levels, measures of glucose tolerance status, and markers of inflammation. Keeping in mind that under the hyperinsulinemic conditions reached during euglycemic hyperinsulinemic clamp studies (with insulin infusion rate fixed 40 mU/m2 per min) hepatic glucose production is suppressed and 80–90% of the infused glucose is taken up by skeletal muscle, insulin-stimulated glucose disposal may be assumed to primarily reflect skeletal muscle insulin sensitivity^13^. Notably we found that IgG2 levels were negatively correlated with both insulin-stimulated glucose disposal and muscle insulin sensitivity index. Accordingly, IgG2 levels were negatively correlated with surrogate OGTT-derived indexes including Stumvoll ISI, Gutt’s ISI_0,120_, Matsuda which are strongly correlated with skeletal muscle insulin sensitivity^14^. By contrast, no significant correlations were found between IgG2 levels and HOMA-IR index, a surrogate index of liver insulin resistance, primarily reflecting hepatic glucose production^14^, and the liver IR index and glucose_0–30_ (AUC) × insulin_0–30_ (AUC) index, which were specifically developed to estimate hepatic insulin resistance^14,15^. These results are in line with animal-based evidence showing that IgG2c affects muscle rather than hepatic insulin sensitivity^12^. To the best of our knowledge, this is the first study in humans assessing the relationship between serum IgG isotypes levels and whole-body insulin sensitivity evaluated using the euglycemic hyperinsulinemic clamp. Preclinical studies have shown that visceral adipose tissue lysates from mice fed with high-fat diet displayed higher concentrations of IgG2c, which are responsible for selective muscle insulin resistance^11,12^, and therefore, it is conceivable that increased adipose tissue mass may explain the relationship between IgG2 and insulin sensitivity. We found that the association between IgG2 levels and whole-body insulin sensitivity remained statistically significant after adjustment for measures of adiposity such as BMI, and waist circumference, it is thus, unlikely that an expansion of fat mass may fully explain this association.

Alternatively, the association between IgG2 and insulin sensitivity may reflect a generalized subclinical inflammatory state rather than a specific effect of IgG2 isotype on insulin sensitivity. However, we found that the association between IgG2 levels and insulin-stimulated glucose disposal remained statistically significant after adjustment for subclinical inflammatory markers such as hsCRP, complement C3 and white blood cell count; this result arguing against the possibility that a dysregulation in the production of inflammatory molecules may explain the relationship between IgG2 and insulin sensitivity. The view that IgG2 is a *bona fide* modulator of insulin sensitivity is also supported by the finding that IgG1, IgG3, and IgG4 are not associated with insulin-stimulated glucose disposal. Moreover previous studies have reported that insulin resistance in obese subjects is associated with a distinct profile of IgG^11^, and that IgG2c and their sialylation levels play a causal role in obesity-induced insulin resistance in mice by modulating insulin endothelial transcytosis via FcγRIIB receptor^12^.

Our study has some strengths including the demographically homogeneous cohort of offspring of patients with type 2 diabetes, equally comprising both male and female subjects, the wealth of exhaustive anthropometric and metabolic variables collected by a trained staff according to an international standardized protocol, the use of the gold standard hyperinsulinemic euglycemic clamp for insulin sensitivity assessment, and the exclusion of confounding conditions potentially affecting both insulin sensitivity and immune response.

Notwithstanding, the current study has a number of potential limitations that should be recognized. First, the present study has a cross-sectional design, and thus it does not allow us to definitely establish the causal relationship between IgG2 levels and muscle insulin sensitivity or to draw any conclusion on the role of IgG2 in the occurrence of insulin resistance characterizing type 2 diabetes, even though prior preclinical evidence supports the biological plausibility of the present findings. Second, the present results might have been influenced by the presence of a family history of type 2 diabetes. Indeed, it has been reported that parental diabetes is associated with a significant increase in total IgG in the offspring^10^. Relatives share not only genetic determinants influencing the immune response but also environmental factors such as exposure to infections. Therefore, the extension of the present results to the general population must be made with caution. Third, the present results are only based on White individuals, and generalizing them to other ethnic groups may be inappropriate, because differences between ethnic groups in concentrations of IgG, IgA, and IgM have been reported^16^. Moreover, we did not have access to direct, detailed measures of hepatic insulin resistance by clamp combined with tracer technique since it is complex and expensive, and therefore not feasible for large epidemiological studies. However, we have employed validated proxy measures of hepatic insulin resistance including the liver IR index and glucose_0–30_ (AUC) × insulin_0–30_ (AUC), which contain more information compared with fasting based measures utilized for the HOMA-IR index^14,15^. Finally, we cannot determine which potential underlying mechanism might induce an elevation in serum IgG2 in insulin resistant individuals. In this regard it should be noted that IgG2 isotype is strongly involved in the responses to bacterial capsular polysaccharide antigens^17^. Accordingly, previous studies have described increased circulating levels of IgG2 in individuals with periodontitis, predominantly reactive against the major oral pathogens, A. actinomycetemcomitans and P. gingivalis^18–20^. On the other hand several evidences have demonstrated the association between periodontal disease and the risk to develop type 2 diabetes supporting the idea that microbial-induced chronic activation of immune response may exert deleterious effects on glucose homeostasis^21–23^. Numerous studies have consistently pointed to a link between stimulation of toll-like receptors (TLRs), by binding of bacterial antigens, activation of pro-inflammatory signaling pathways and insulin resistance^24–26^. Notably TLRs not only play a critical role in innate immunity but are also essential in the modulation of humoral responses^27,28^. In particular, TLR-4 activated by bacterial lipopolysaccharide (LPS) elicits IgG2 response by promoting Th1 associated cytokines production such as interleukin-12 (IL-12) and interferon gamma (IFN-γ)^29,30^. Importantly, alteration of gut microbiota or intestinal permeability, as observed in insulin resistance-related conditions, has been shown to increase gut-derived LPS circulating levels, thus resulting in systemic activation of TLR4-driven immune response^25,31,32^.

Considering these data we can hypothesize that all these mechanisms may be responsible for increased production of IgG2, which, in turn, leads to insulin resistance, however we cannot exclude that the increased levels of IgG2 found in subjects with a higher degree of insulin resistance represent an epiphenomenon of other alterations, directly involved in the pathogenesis of insulin resistance.

In conclusion the present findings indicate that serum concentrations of IgG2, but not IgG1, IgG3 or IgG4, are associated with insulin-stimulated glucose disposal. The current study is novel because it suggests that the relationship between IgG2 isotype and insulin sensitivity is independent of important determinants of glucose metabolism including gender, age, adiposity, lipids, and subclinical inflammation. Future studies are needed to examine whether IgG2 plays a specific role in the development of type 2 diabetes independently of confounders factors.

## Materials and Methods

The study **s**ample comprised 262 non-diabetic offspring of patients with type 2 diabetes participating in the European Network on Functional Genomics of Type 2 Diabetes (EUGENE2) project^33^. Exclusion criteria included: history of type 1 and type 2 diabetes, malignant diseases, inflammatory bowel diseases and gastrointestinal disorders associated with malabsorption, chronic pancreatitis, use of drugs able to interfere with glucose homeostasis and levels of inflammatory markers such as steroids and oral contraceptive agents, history of alcohol abuse, immunological or rheumatic diseases, acute and chronic infections or positivity for antibodies to hepatitis C virus (HCV) or hepatitis B surface antigen (HBsAg). Individuals were consecutively recruited at the Department of Medical and Surgical Sciences of the University ‘Magna Graecia’ of Catanzaro as previously described^33,34^. All subjects were of European ancestry and underwent anthropometrical evaluation including measurements of body mass index (BMI), waist circumference, and body composition assessed by bioelectrical impedance. A 75 g oral glucose tolerance test (OGTT) was performed with 0, 30, 60, 90 and 120 min sampling for plasma glucose and insulin assays. Insulin sensitivity was determined by euglycemic hyperinsulinemic clamp study, as previously described^34^. Briefly, a priming dose of insulin (Humulin, Eli Lilly & Co., Indianapolis, IN) was administered during the initial 10 min to acutely raise plasma insulin followed by continuous insulin infusion fixed at 40 mU/m^2^ × min. The blood glucose level was maintained constant during the 2-h clamp study by infusing 20% glucose at varying rates according to blood glucose measurements assessed by a glucose analyzer at 5 minute intervals (mean coefficient of variation of blood glucose was <5%).

The study was approved by the local ethics committee (Comitato Etico Azienda Ospedaliera “Mater Domini”). Written informed consent was obtained from each participant before commencing the study, in accordance with the principles of the Declaration of Helsinki.

### Analytical determinations

Glucose, triglycerides, total and high density lipoprotein (HDL) cholesterol concentrations were determined by enzymatic methods (Roche, Basel, Switzerland). Serum insulin concentrations were determined with a chemiluminescence-based assay (Immulite®, Siemens, Italy). White blood cell count was determined using an automated particle counter (Siemens Healthcare Diagnostics ADVIA® 120/2120 Hematology System, Italy). Serum levels of high sensitivity C reactive protein (hsCRP), complement C3, total IgG, IgG1, IgG2, IgG3 and IgG4 subclasses were assayed by an automated nephelometric technology using the BN™II System analyzer with high levels of sensibility and specificity (Siemens Healthcare, Italy). Glycated hemoglobin (HbA1c) was assessed by high performance liquid chromatography employing a National Glycohemoglobin Standardization Program certified automated analyzer (Adams HA-8160 HbA1C analyzer, Menarini, Italy).

### Calculations

Glucose disposal was calculated as the mean rate of glucose infusion measured during the last 60 min of the clamp examination (steady-state) and it is expressed as milligrams per minute per kilogram fat-free mass measured with the use of electrical bioimpedance. The HOMA-IR index was calculated as fasting insulin × fasting glucose/22.5^35^. The liver IR index was calculated using the formula: −0.091 + (log insulin area under the curve [AUC] 0–120 min × 0.400) + (log fat mass % × 0.346) − (log HDL Cholesterol × 0.408) + (log BMI × 0.435)^15^. The glucose_0–30_ (AUC) × insulin_0–30_ (AUC) index of hepatic resistance to insulin was calculated as reported^14^. The muscle insulin sensitivity index was calculated as the rate of decline in plasma glucose concentration divided by mean plasma insulin concentration during OGTT as described by Abdul-Ghani MA *et al*.^14^. The QUICKI index was calculated using the formula: 1/(log insulin_0_ + log glucose_0_) as reported^36^. The Stumvoll ISI_OGTT_ was calculated as 0.226 − 0.0032 × BMI − 0.0000645 × Insulin_120_ − 0.00375 × Glucose_90_^37^. The Matsuda index was calculated as 10,000/square root of [fasting glucose (mmol/L) × fasting insulin (mU/L)] × [mean glucose × mean insulin during OGTT]^38^. The Gutt’s ISI_0,120_ index insulin sensitivity was calculated using the formula: (m/[(G_0_ + G_120_)/2])/log [(I_0_ + I_120_)/2] where m = (75,000 mg + (fasting glucose − 2-h glucose) × 0.19 × body weight)/120 min (glucose in mg/dl; insulin in µIU/ml) as previously described^39^.

### Statistical analysis

The results for continuous variables are given as means ± SD. Variables with skewed distribution including triglycerides, fasting insulin, HOMA-IR index, glucose_0–30_ (AUC) × insulin_0–30_ (AUC) index, Gutt’s ISI_0,120_, Stumvoll ISI_OGTT_, Matsuda, muscle insulin sensitivity index and hsCRP were natural log transformed for statistical analyses. Unpaired Student’s t was used to compare differences of continuous variables between two groups. Relationships between variables were determined by Pearson’s correlation coefficient (*r*). Partial correlation coefficients adjusted for age and gender were computed between variables. A multivariable linear regression analysis was performed in order to evaluate the independent contribution of IgG isotypes and other inflammatory and metabolic variables to insulin sensitivity. The variance inflection factor (VIF) was less than 2 in all the analyses indicating that multicollinearity among variables was not a problem in the multiple regression models. The normality of the standardized residuals was tested using the Kolmorogov-Smirnov test that was >0.05 indicating a normal distribution of the residuals in the multiple regression model.

All tests were two-sided, and a P value < 0.05 was considered statistically significant. All analyses were performed using SPSS (Chicago, IL, USA) software programme Version 22.0 for Windows.

### Ethical approval and informed consent statement

The study was approved by the local ethics committee (Comitato Etico Azienda Ospedaliera “Mater Domini”). Written informed consent was obtained from each study participant. All procedures were performed in accordance with the principles of the Declaration of Helsinki.

## Electronic supplementary material

Supplementary Information
