# Supplementary material for: Serum IgG2 levels are specifically associated with whole-body insulin-mediated glucose disposal in non-diabetic offspring of type 2 diabetic individuals: a cross-sectional study
Source: Sci Rep. 2018 Sep 11;8:13616. doi: 10.1038/s41598-018-32108-8 (PMC6134058; doi:10.1038/s41598-018-32108-8)
Supplement: Supplementary file 1 — Supplementary Information [file 41598_2018_32108_MOESM1_ESM.pdf]

Serum IgG2 levels are specifically associated with whole-body insulin-mediated glucose disposal in non-diabetic offspring of type 2 diabetic individuals: a cross-sectional study

Teresa Vanessa Fiorentino[a] , Elena Succurro[a], Franco Arturi[a], Aida Giancotti[b], Cinzia Peronace[b], Angela Quirino[b], Franz Sesti[c], Francesco Andreozzi[a], Marta Letizia Hribal[a], Francesco Perticone[a], Alfredo Focà[b], Giorgio Sesti*[a]

[a]Department of Medical and Surgical Sciences, University Magna Graecia of Catanzaro, Viale Europa, Catanzaro, 88100, Italy.

[b]Department of Health Sciences, "Magna Græcia" University of Catanzaro, Viale Europa, 88100 Catanzaro, Italy.

[c]Department of Experimental Medicine, Sapienza University of Rome, Rome, 00161, Italy

Suppl Table 1 – Age and gender adjusted univariate correlations between IgG isotypes levels and anthropometric and metabolic variables in individuals with normal glucose tolerance

	**Total IgG (*mg/ml*)**		**IgG1 (*mg/ml*)**		**IgG2 (*mg/ml*)**		**IgG3 (*mg/ml*)**		**IgG4 (*mg/ml*)**	
	**Pearson's correlation coefficient (*r*)**	***P* value**	**Pearson's correlation coefficient (*r*)**	***P* value**	**Pearson's correlation coefficient (*r*)**	***P* value**	**Pearson's correlation coefficient (*r*)**	***P* value**	**Pearson's correlation coefficient (*r*)**	***P* value**
Age *(yrs)*	0.02	0.78*	-0.008	0.90*	0.41	0.56*	0.02	0.78*	-0.09	0.17*
BMI *(kg/m^2)*	0.05	0.22	0.07	0.18	0.06	0.19	-0.06	0.18	-0.03	0.33
Waist circumference (*cm*)	0.009	0.45	0.02	0.40	0.04	0.28	-0.07	0.15	-0.05	0.24
Fat body mass (*%*)	0.08	0.24	0.10	0.16	0.01	0.86	-0.04	0.53	0.01	0.86
Fat free mass (*%*)	-0.06	0.40	-0.08	0.25	0.008	0.90	0.06	0.42	0.01	0.89
Systolic blood pressure *(mmHg)*	0.08	0.12	0.05	0.24	0.01	0.44	0.07	0.16	0.05	0.25
Diastolic blood pressure *(mmHg)*	**0.15**	**0.02**	0.08	0.11	0.01	0.41	0.07	0.13	0.10	0.07
Total Cholesterol *(mg/dl)*	0.02	0.36	0.07	0.13	-0.01	0.48	0.07	0.13	0.02	0.22
HDL Cholesterol *(mg/dl)*	-0.7	0.17	-0.08	-0.12	-0.06	0.19	-0.10	0.08	-0.06	0.17
Triglycerides *(mg/dl)*	0.08	0.25	**0.14**	**0.05**	0.07	0.30	**0.15**	**0.03**	0.08	0.22
Fasting Glucose *(mg/dl)*	0.10	0.16	0.08	0.26	0.03	0.67	-0.06	0.35	0.02	0.77
2-h glucose (*mg/dl*)	0.02	0.76	0.009	0.89	0.3	0.70	-0.07	0.33	-0.05	0.44
HbA1c (%) [*mmol/mol*]	-0.12	0.22	-0.07	0.50	-0.07	0.49	0.005	0.95	0.02	0.80
hsCRP (*mg/l*)	0.03	0.71	-0.03	0.68	0.06	0.44	-0.06	0.37	-0.01	0.80

Complement C3 (*g/l*)	**0.22**	**0.007**	0.14	0.04	0.10	0.09	0.05	0.26	0.03	0.36
White blood cell count (*cell/mm*^{3})	0.02	0.37	-0.08	0.11	0.08	0.12	-0.02	0.39	-0.03	4534
Insulin-stimulated glucose disposal ($mg * min^{-1} * kg\ FFM^{-1}$)	-0.04	0.28	-0.02	0.36	**-0.14**	**0.02**	-0.05	0.25	-0.008	0.34
HOMA-IR	0.08	0.13	0.07	0.14	0.07	0.15	0.01	0.41	-0.03	0.32
Liver IR index	0.12	0.12	0.11	0.15	0.10	0.19	0.13	0.08	-0.04	0.58
QUICKI	-0.09	0.21	-0.08	0.26	-0.07	0.32	-0.007	0.91	0.03	0.68
Glucose $_{0–30}$ (AUC) x insulin$_{0–30}$ (AUC)	0.05	0.51	0.09	0.25	0.005	0.95	0.12	0.09	-0.07	0.38
Stumvoll ISI$_{OGTT}$	-0.12	0.07	-0.01	0.46	**-0.21**	**0.008**	0.007	0.46	-0.008	0.46
Matsuda index	-0.13	0.04	-0.11	0.07	**-0.13**	**0.05**	-0.08	0.14	0.01	0.40
Gutt's ISI$_{0,120}$ index	-0.13	0.04	-0.12	0.09	-0.11	0.16	-0.05	0.49	0.02	0.82
Muscle insulin sensitivity index	**-0.18**	**0.04**	-0.09	0.27	**-0.18**	**0.03**	-0.15	0.09	0.45	0.61

**P* values refer to results after analyses with adjustment for gender. Triglycerides, HOMA-IR index, glucose $_{0–30}$ (AUC) x insulin$_{0–30}$ (AUC), Stumvoll ISI$_{OGTT}$, Matsuda, Gutt's ISI$_{0,120}$, Muscle insulin sensitivity index, and hsCRP levels were log transformed for statistical analysis.

BMI = body mass index; HbA1c = glycated hemoglobin; hsCRP = high sensitivity C reactive protein; HDL = high density lipoprotein; HOMA-IR = homeostasis model assessment insulin resistance; Liver IR = liver insulin resistance.

Suppl. Table 2 – Age and gender adjusted univariate correlations between IgG isotypes levels and anthropometric and metabolic variables in individuals with prediabetes

	**Total IgG (*mg/ml*)**		**IgG1 (*mg/ml*)**		**IgG2 (*mg/ml*)**		**IgG3 (*mg/ml*)**		**IgG4 (*mg/ml*)**	
	**Pearson's correlation coefficient (*r*)**	***P* value**	**Pearson's correlation coefficient (*r*)**	***P* value**	**Pearson's correlation coefficient (*r*)**	***P* value**	**Pearson's correlation coefficient (*r*)**	***P* value**	**Pearson's correlation coefficient (*r*)**	***P* value**
Age *(yrs)*	-0.09	0.48*	-0.15	0.21*	-0.07	0.55*	-0.02	0.89*	0.05	0.72*
BMI *(kg/m^2)*	0.13	0.29	0.18	0.16	0.10	0.45	0.11	0.39	0.08	0.53
Waist circumference (*cm*)	0.16	0.21	0.22	0.09	0.09	0.47	0.05	0.68	0.04	0.73
Fat body mass (*%*)	0.04	0.74	0.03	0.84	0.04	0.78	-0.09	0.49	0.01	0.92
Fat free mass (*%*)	-0.22	0.08	-0.14	0.27	-0.01	0.91	0.001	0.99	-0.01	0.90
Systolic blood pressure *(mmHg)*	0.006	0.96	-0.13	0.31	-0.03	0.82	-0.16	0.22	-0.03	0.76
Diastolic blood pressure *(mmHg)*	0.06	0.66	-0.02	0.88	-0.06	0.64	-0.04	0.76	0.06	0.65
Total Cholesterol *(mg/dl)*	-0.09	0.48	-0.16	0.22	-0.07	0.59	-0.04	0.78	-0.12	0.36
HDL Cholesterol *(mg/dl)*	-0.17	0.19	**-0.26**	**0.04**	-0.06	0.62	-0.15	0.25	-0.14	0.28
Triglyceride *(mg/dl)*	0.05	0.68	-0.007	0.95	-0.07	0.58	0.07	0.55	-0.08	0.50
Fasting Glucose *(mg/dl)*	-0.05	0.68	-0.04	0.70	0.02	0.87	-0.16	0.11	-0.02	0.89
2-h glucose (*mg/dl*)	0.13	0.39	0.07	0.58	0.20	0.07	0.07	0.59	0.05	0.69
HbA1c (%) [*mmol/mol*]	-0.20	0.31	-0.12	0.54	-0.26	0.19	0.07	0.73	0.01	0.95
hsCRP (*mg/l*)	0.009	0.94	0.11	0.43	0.06	0.65	0.14	0.30	-0.06	0.62

Complement C3 (*g/l*)	0.03	0.82	-0.07	0.64	0.03	0.83	0.22	0.13	-0.09	0.55
White blood cell count (*cell/mm*^{3})	-0.03	0.79	0.03	0.80	0.16	0.11	0.19	0.08	-0.05	0.69
Insulin-stimulated glucose disposal (*mg* $*$ *min*$^{-1}$ $*$ *kg FFM*$^{-1}$)	-0.13	0.15	0.03	0.39	**-0.24**	**0.03**	-0.05	0.34	-0.14	0.14
HOMA-IR	0.03	0.81	0.001	0.98	0.11	0.37	0.16	0.20	0.13	0.31
Liver IR index	0.12	0.40	0.14	0.31	0.04	0.77	0.07	0.65	0.09	0.52
QUICKI	-0.03	0.82	0.06	0.62	-0.13	0.32	0.20	0.12	-0.13	0.31
Glucose $_{0–30}$ (AUC) x insulin$_{0–30}$ (AUC)	0.06	0.34	0.005	0.48	-0.04	0.39	0.09	0.27	0.10	0.24
Stumvoll ISI$_{OGTT}$	-0.03	0.42	-0.08	0.28	-0.12	0.21	0.02	0.45	-0.12	0.21
Matsuda index	-0.10	0.47	0.07	0.31	-0.05	0.35	0.12	0.19	-0.12	0.20
Gutt's ISI$_{0,120}$ index	-0.12	0.19	0.04	0.38	-0.19	0.08	0.05	0.36	-0.14	0.15
Muscle insulin sensitivity index	-0.13	0.46	-0.03	0.87	-0.15	0.42	-0.10	0.58	-0.008	0.96

**P* values refer to results after analyses with adjustment for gender. Triglyceride, HOMA-IR index, glucose $_{0–30}$ (AUC) x insulin$_{0–30}$ (AUC), Stumvoll ISI$_{OGTT}$, Matsuda, Gutt's ISI$_{0,120}$, Muscle insulin sensitivity index and hsCRP levels were log transformed for statistical analysis.

BMI = body mass index; HbA1c = glycated hemoglobin; hsCRP = high sensitivity C reactive protein; HDL = high density lipoprotein; HOMA-IR = homeostasis model assessment insulin resistance; Liver IR = liver insulin resistance.
